# Hepatotoxicity in Mice of a Novel Anti-parasite Drug Candidate Hydroxymethylnitrofurazone: A Comparison with Benznidazole

**DOI:** 10.1371/journal.pntd.0003231

**Published:** 2014-10-16

**Authors:** Carolina Davies, Nilay Dey, Olga Sanchez Negrette, Luis Antonio Parada, Miguel A. Basombrio, Nisha Jain Garg

**Affiliations:** 1 Instituto de Patología Experimental, Universidad Nacional de Salta-CONICET, Salta, Argentina; 2 Department of Microbiology and Immunology, University of Texas Medical Branch, Galveston, Texas, United States of America; 3 Cátedra de Quimica Biológica, Facultad de Ciencias Exactas, Universidad Nacional de Salta, Argentina; 4 Department of Pathology, University of Texas Medical Branch, Galveston, Texas, United States of America; 5 Institute for Human Infections and Immunity, University of Texas Medical Branch, Galveston, Texas, United States of America; Northeastern University, United States of America

## Abstract

**Background:**

Treatment of Chagas disease, caused by *Trypanosoma cruzi*, relies on nifurtimox and benznidazole (BZL), which present side effects in adult patients, and natural resistance in some parasite strains. Hydroxymethylnitrofurazone (NFOH) is a new drug candidate with demonstrated trypanocidal activity; however, its safety is not known.

**Methods:**

HepG2 cells dose response to NFOH and BZL (5–100 µM) was assessed by measurement of ROS, DNA damage and survival. Swiss mice were treated with NFOH or BZL for short-term (ST, 21 d) or long-term (LT, 60 d) periods. Sera levels of cellular injury markers, liver inflammatory and oxidative stress, and fibrotic remodeling were monitored.

**Results:**

HepG2 cells exhibited mild stress, evidenced by increased ROS and DNA damage, in response to NFOH, while BZL at 100 µM concentration induced >33% cell death in 24 h. In mice, NFOH ST treatment resulted in mild-to-no increase in the liver injury biomarkers (GOT, GPT), and liver levels of inflammatory (myeloperoxidase, TNF-α), oxidative (lipid peroxides) and nitrosative (3-nitrotyrosine) stress. These stress responses in NFOH LT treated mice were normalized to control levels. BZL-treated mice exhibited a >5-fold increase in GOT, GPT and TNF-α (LT) and a 20–40% increase in liver levels of MPO activity (ST and LT) in comparison with NFOH-treated mice. The liver inflammatory infiltrate was noted in the order of BZL>vehicle≥NFOH and BZL>NFOH≥vehicle, respectively, after ST and LT treatments. Liver fibrotic remodeling, identified after ST treatment, was in the order of BZL>vehicle>NFOH; lipid deposits, indicative of mitochondrial dysfunction and in the order of NFOH>vehicle>BZL were evidenced after LT treatment.

**Conclusions:**

NFOH induces mild ST hepatotoxicity that is normalized during LT treatment in mice. Our results suggest that additional studies to determine the efficacy and toxicity of NFOH are warranted.

## Author Summary

Hydroxymethylnitrofurazone (NFOH) is a promising drug candidate with demonstrated trypanocidal activity in experimental models of *Trypanosoma cruzi* infection and chronic disease development. In this study, we monitored the safety of NFOH in established *in vitro* and *in vivo* models. Our data show that NFOH did not induce hepatocyte cell death. Short-term or long-term treatment of mice with NFOH did not induce hepatic stress measured by cellular injury, inflammation or fibrosis. Benznidazole, the currently used treatment against acute infection in humans, was more toxic and induced chronic inflammation and liver injury in mice. We conclude that NFOH should be studied further to determine its potential safety for human use as an anti-parasite therapy.

## Introduction

Chagas disease is endemic in 21 countries, and the World Health Organization estimates that approximately 9-million people are infected by *Trypanosoma cruzi* [1]. The acute phase lasts for ∼2-months, and is characterized by high parasitemia and fever. Chronic chagasic cardiomyopathy is the most severe clinical consequence of *T. cruzi* infection, which is detectable in 30% of the patients several years after the primary infection [2].

A considerable advancement in the knowledge about the biology of the *T. cruzi* parasite has been made in the last few decades (reviewed in [3]). Yet, treatment of Chagas disease still relies on two drugs, namely nifurtimox (NFX, Bayer) and benznidazole (BZL), that were developed during the 1960s and are currently manufactured and distributed by Roche (Rochagan, in Brazil), and Maprimed and ELEA laboratories (Abarax) in Argentina [4]. Since the *T. cruzi* life cycle involves intracellular division and subsequent parasite release to peripheral blood, a long-term treatment period (usually an oral dose for 60 days) is required. Both drugs are well tolerated by infants and used as standard treatment to control acute infection by *T. cruzi* in children [5]. However, chronic chagasic cardiomyopathy is a complex malady that involves immune mechanisms as well as parasite persistence. Several studies have noted that treatment with BZL or NFX is not always effective in controlling the chronic disease in chagasic patients. Moreover, these drugs trigger multiple side effects in adults, resulting in noncompliance with long-term treatment [6]. Further, BZL and NFX are not effective against all naturally occurring *T. cruzi* strains, and some strains have been documented to acquire resistance to these drugs [7]–[9]. Thus, alternative therapeutic drugs that are well-tolerated, safe, and effective against *T. cruzi* are urgently needed.

Due to their limited commercial potential, development of new drugs for Chagas disease is perhaps not a viable option. Instead, drug repurposing–finding a new indication for an existing drug—has enormous potential in developing a new therapy against parasitic diseases. Nitrofurazone (NF), commercialized as a topical medicine for bactericidal activity against gram-positive and gram-negative bacteria, has recently been shown to inhibit trypanothione reductase, the main enzyme responsible for xenobiotic metabolism in *T. cruzi* [10]. Subsequently, NF was found to have significant anti-*T. cruzi* activity [11]. Unfortunately, NF showed toxicity against mammalian cells [12] and long-term treatment with NF induced ovarian cancer in mice and rats [13], [14], spurring the identification of alternative chemicals with specific activity against *T. cruzi* only. Among the latter, hydroxymethylnitrofurazone (NFOH) was identified as a derivative of NF. The reduction of nitrofurazone is pH-dependent and in acidic medium the hydroxylamine derivative, involving four electrons, is the principal product formed. In aqueous-alkaline medium, the reduction of nitrofurazone occurs in two steps, the first involving one electron to form the nitro-radical anion and the second corresponding to the hydroxylamine derivative formation. NFOH presented the same voltammetric behavior and electroactivity, indicating that the molecular modification performed in NF did not change its capacity to be reduced [15]. We and others have shown NFOH has 2-fold more cytotoxic activity than the parental compound NF against *T. cruzi* [15], [16]. The mechanism of action of NFOH against *T. cruzi* is not completely clear; however, like all nitroheterocyclic compounds, it is enzymatically reduced at the nitro group resulting in the generation of nitroanion (RNO_2_^•−^) and hydronitroxide (RNHO^•−^) free radicals [17]. NFOH has also been shown to at least partially interfere with mRNA trans-splicing [16] and cruzipain activity [18] that are essential for parasite invasion as well as differentiation to replicative form.

Before NFOH can be tested and promoted further as an anti-*T. cruzi* drug for human use, it is essential that we evaluate its safety profile. In a therapeutic regimen administered to *T. cruzi* infected mice, NFOH and BZL provided comparable control of *T. cruzi* and survival from infection (84% and 67%, respectively) [11], while NF caused 75% mortality in infected mice. Accordingly, in this study, we have evaluated the liver toxicity of NFOH in comparison with BZL by using *in vitro and in vivo* models. We treated HepG2 liver cells and mice with the two drugs and assessed inflammation, oxidative stress, and cell survival or tissue remodeling. We chose to treat mice with NFOH for short-term (ST) and long-term (LT) periods that were similar to the recommended regimen for the BZL treatment of children and adults exposed to *T. cruzi* infection.

## Materials and Methods

### Ethics statement

All animal experiments were performed according to the National Institutes of Health Guide for Care and Use of Experimental Animals and approved by the Ethical Committee of the National University of Salta and the Animal Care and Use Committee at the UTMB (protocol # 08-05-029).

### Cell, mice and treatment

A hepG2 human hepatocyte cell line was obtained from the American Tissue Culture Collection (Maryland, USA). The cells were cultured in complete DMEM high glucose media (Gibco) supplemented with 10% FBS at 37°C, 5% CO_2_.

Female Swiss mice were bred at the Instituto de Patologia Experimental mouse facility. Swiss mice have been widely employed as animal models for experimental chemotherapy in Chagas disease [19]. NFOH was kindly provided by Dr. Man Chin Chung (Faculdade de Ciencias Farmaceuticas, Universidade Estadual Paulista, Brazil). BZL (Roche Pharmaceuticals, Brazil) was obtained from the Ministry of Public Health, Province of Salta, Argentina. Mice (30-day old) received NFOH (150 mg/kg/day) or BZL (150 mg/kg/day), suspended in 9% NaCl/5% Tween-80 (vehicle solution), as an oral dose of 100 µl, once a day (six days per week). The selected doses of the BZL and NFOH mimicked the dose per kg and dose regime (two months of daily treatment) of humans. All experiments were carried out in female mice because they develop disease symptoms similar to those seen in human infection. Additionally, we have noted that the female mice exhibited a higher tolerance to infection than did male mice [11]. One set of mice (n = 9/group) received the treatment for 21 days to allow us to determine the acute (short-term, ST) liver toxicity of the drugs. Another set of mice (n = 9/group) received the treatment for 60 days to facilitate our knowledge of the chronic (long-term, LT) liver toxicity of the drugs. Mice given the vehicle solution were used as controls. After treatment, mice were sacrificed, and sera samples and liver tissues stored at −80°C until use.

### Cell survival and proliferation

HepG2 cells were cultured as above, seeded at 7.5×10^4^ cells/well in 96-well microplate, and incubated overnight in complete media at 37°C, 5% CO_2_. Cells were treated in serum-free medium with NFOH or BZL (5, 50 and 100 µM) for 24–48 h. To examine the drug-induced changes in cell viability and proliferation, after the drug treatment, we incubated the cells for 30 min in the presence of AlamarBlue reagent (Life Technologies/Invitrogen, 10% final concentration). Resazurin, the active ingredient of alamarBlue reagent, is a non-toxic, cell-permeable compound that is blue in color and virtually non-fluorescent. Upon entering cells, resazurin is reduced to resorufin, a compound that is red in color and highly fluorescent. Viable cells continuously convert resazurin to resorufin, increasing the overall fluorescence and color of the media surrounding cells. Fluorescence was measured at Ex_540_/Em_590_ nm on a SpectraMax Microplate Reader. Results were analyzed as per the manufacturer's instructions.

### Flow cytometry analysis of ROS production and DNA damage

HepG2 cells were incubated in the presence or absence of NFOH or BZL for 24 h, as above. For the quantitation of reactive oxygen species (ROS), 5 µM CellROX Green reagent was added to each well in complete media, and cells were incubated at 37°C for 30 minutes. CellROX Green is cell-permeant and non-fluorescent, or very weakly fluorescent, in the reduced state. Upon oxidation, the reagents exhibit strong fluorescence and remain localized within the cell. Cells were washed with PBS and analyzed by flow cytometry.

For assessing the effect of NFOH and BZL in inducing DNA damage, HepG2 cells were treated with NFOH or BZL for 24 h, as above. Cells were harvested, washed with PBS, fixed with 3.7% paraformaldehyde for 15 min at 4°C, and permeabilized with 90% methanol. Cells were then incubated at room temperature for 2 h with mouse anti-8-oxo-dG antibody (250-fold dilution, EMD Milipore, Billerica, MA) and for 30 min with PE-conjugated, anti-mouse IgG (eBioscience, San Diego, CA). Cells stained with isotype-matched IgGs were used as controls. Samples were visualized on an LSRII Fortessa Cell Analyzer, acquiring 30–50,000 events in a live cell gate, and further analysis performed by using FlowJo software (version 7.6.5, Tree-Star, San Carlo, CA).

### Tissue homogenates

Frozen liver tissues (25 mg) were homogenized in 0.5 ml of ice-cold lysis buffer (25 mM Tris pH 7.6, 150 mM NaCl, 1% sodium deoxycholate, 1% Igepal CA-630, 0.1% SDS, 10 µl/ml sodium orthovanadate, 10 mM PMSF and 10 µl/ml Sigma protease inhibitor cocktail), and centrifuged at 3000 g for 10 min at 4°C. Supernatants were stored at −80°C, and protein concentration determined by the Bradford method.

### Cellular injury markers

The activities of glutamate oxaloacetate transaminase (GOT) and glutamate pyruvate transaminase (GPT), alternatively called aspartate transaminase (AST) and alanine transaminase (ALT), respectively, were determined by using commercially available assay kits (Wiener Lab, Rosario, Argentina). Briefly, for the GOT/AST assay, 50 µl of sera sample or liver homogenate (∼100-µg protein) was added to reagent A containing 12 mM 2-oxoglutarate, 0.18 mM NADH, 420 U/l malate dehydrogenase (MDH), and 600 U/l lactate dehydrogenase (LDH). The reaction was started by adding 80 mM Tris HCl buffer, pH 7.8, containing 240 mM L-aspartate, and the resultant oxaloacetate formation coupled with NADH oxidation by MDH monitored at 340 nm. For the GPT/ALT assay, 50 µl of sample was added to reagent A containing NADH, 2-oxoglutarate (as above) and 1200 U/l LDH. The reaction was started by adding 80 mM Tris HCl buffer, pH 7.8, containing 500 mM L-alanine, and resultant reduction of pyruvate coupled with NADH oxidation by LDH monitored at 340 nm (ε = 6,220 M^−1^cm^−1^).

### Oxidative/nitrosative stress

We measured lipid peroxides, a biomarker of oxidative stress [20], by using a LPO Assay Kit (Cayman). Briefly, liver homogenate LPOs were extracted into chloroform, mixed with methanol (1∶1, v/v), and added in triplicate (55 µl/well) to 96-well plates. The reaction was started with addition of 50 µl/well of 4.5 mM FeSO_4_/0.2 M HCl, 3% ammonium thiocyanate (chromogen) solution. The redox reaction with ferrous ions was stopped after 5 min, and absorbance monitored at 500 nm (standard curve: 0–500 µM 13-hydroperoxy octadecadienoic acid).

The level of protein nitrosylation, an indicator of nitrosative stress, was determined by Western blotting [21]. Tissue homogenates (10-µg protein) were resolved on 10% SDS polyacrylamide gels, and transferred to PVDF membranes by using a vertical Criterion Blotter (Bio-Rad). Membranes were washed in TBS (20.4 mM Tris, 150 mM NaCl, pH 7.6), blocked for 1 h in 5% nonfat milk (NFM), and incubated overnight at 4°C with anti-3-nitrotirosine antibody (clone 2A8.2, 1∶2000, Millipore). After washing with TBS-T (TBS/0.1% Tween-80), membranes were incubated for 1 h at room temperature with HRP-conjugated secondary antibody (1∶10,000, Southern Biotech), and signal was developed with an enhanced chemiluminiscence detection system (GE-Healthcare). Membranes were incubated in stripping buffer (Thermo Scientific) and probed with anti-β-actin antibody (1∶10,000, Sigma) to confirm an equal loading of samples. All antibody dilutions were made in NFM. Spot densitometry for protein bands was carried out using a FluorChem HD2 Image Analyzer (Alpha Innotech).

### Myeloperoxidase

MPO activity was determined as a biomarker of macrophage/neutrophil activation [20]. Liver homogenates (10 µg protein) were added in triplicate to 0.53 mM o-dianisidine dihydrochloride and 0.15 mM H_2_O_2_ in 50 mM KH_2_PO_4_/K_2_HPO_4_ buffer (pH 6.0). After incubation for 5 min at room temperature, the reaction was stopped with 30% sodium azide, and the change in absorbance was measured at 460 nm. Sample protein content was measured by the Bradford Method, and 1 unit MPO was defined as that degrading 1 n mol H_2_O_2_/min at 25°C (ε = 11300 M^−1^.cm^−1^).

### Cytokine levels

*TNF-α* levels were measured as a molecular marker of inflammation. Total RNA was isolated from frozen tissue sections by using the RNeasy plus Kit (Qiagen), and analyzed for quality and quantity on a SpectraMax UV microplate reader. After reverse transcription of 2 µg RNA with poly(dT)_18_, first-strand cDNA was used as a template in a real-time PCR on an iCycler Thermal Cycler with SYBR-Green Supermix (Bio-Rad) and specific oligonucleotides for *TNF-α* (5′-GTT CTA TGG CCC AGA CCC TCA CA-3′ and 5′-TAC CAG GGT TTG ACC TCA GC-3′) and *GAPDH* (5′-TGG CAA AGT GGA GAT TGT TG-3′ and 5′-TTC AGC TCT GGG ATG ACC TT-3′). The PCR Base Line Subtracted Curve Fit mode was applied for Threshold Cycle (C_t_) and mRNA level measured by iCycler iQ Real-Time Detection Software (Bio-Rad). The threshold cycle (*C_t_*) values for target mRNA were normalized to *GAPDH* mRNA, and the relative expression level of *TNF-α* gene was calculated with the formula *n*-fold change = 2^−Δ*Ct*^, where Δ*C_t_* represents *C_t_* (TNF-α)−*C_t_* (GAPDH) [22].

Tissue homogenates were also subjected to measurement of TNF-α cytokine by using an optEIA ELISA kit (Pharmingen, San Diego, CA).

### Histology

Liver tissues were fixed in formalin, embedded in paraffin, and 5-µm sections were stained with hematoxylin and eosin (H&E) and Masson's Trichrome to examine inflammatory infiltrates and collagen deposition, respectively. Cryostat tissue-sections (fixed in OCT cryostat-embedding medium, TissueTek) were stained with Oil red O to examine lipid/fat deposition.

In general, we analyzed each tissue section for >10-microscopic fields (100× magnification), and examined three different tissue sections/mouse (n = 3–4 mice/group) to obtain a semi-quantitative score. Presence of inflammatory cells was scored as I (absent), II (focal or mild, 0–1 foci), III (moderate, ≥2 foci), IV (extensive inflammatory foci, minimal necrosis, and retention of tissue integrity), and V (diffused inflammation with severe tissue necrosis, interstitial edema, and loss of integrity). Inflammatory infiltrates were characterized as diffused or focal depending upon how closely the inflammatory cells were associated. Fibrosis and lipid deposition were assessed by measuring the Masson's Trichrome-stained collagen area (blue) and Oil Red O-stained intrahepatocyte lipid area (red), respectively, as a percentage of the total area by using Simple PCI software (version 6.0; Compix, Sewickley, PA) connected to an Olympus polarizing microscope system (Center Valley, PA). All pixels with blue stain in Masson's trichrome-stained sections and red stain in Oil Red O were selected to build a binary image, and utilized for calculating the percentage of the area occupied by collagen and lipid droplets, respectively. The fibrotic area was further scored as I (<10% of total area), II–III (10–30% of total area), III–IV (30–60% of total area) and V (>60% of total area). Oil red O (intrahepatocyte lipid deposition) was scored as I (absent), II (<10% of total area or patchy distribution of tiny red droplets), III (10–30% of total area or scattered tiny red droplets), and IV (>30% of total area or intense red staining of variable size droplets) [23].

### Data analysis

Data (mean ± SD) were derived from at least triplicate observations per sample (n = 9–12 animals/group), confirmed to be normally distributed by a Q-Q test and histogram plot, and analyzed by Student's *t* test (comparison of two-groups) and 1-way analysis of variance (ANOVA) with a Holm-Sidak test (comparison of multiple groups). Non-parametric Kruskal-Wallis Dunn's test was used to analyze the statistical significance for each cytokine's gene expression. The level of significance is presented by ^*^ (normal versus treated; *p<0.05, ^**^ *p*<0.01).

## Results

### *In vitro* cytotoxicity of NFOH in HepG2 cells

**Figure 1:**
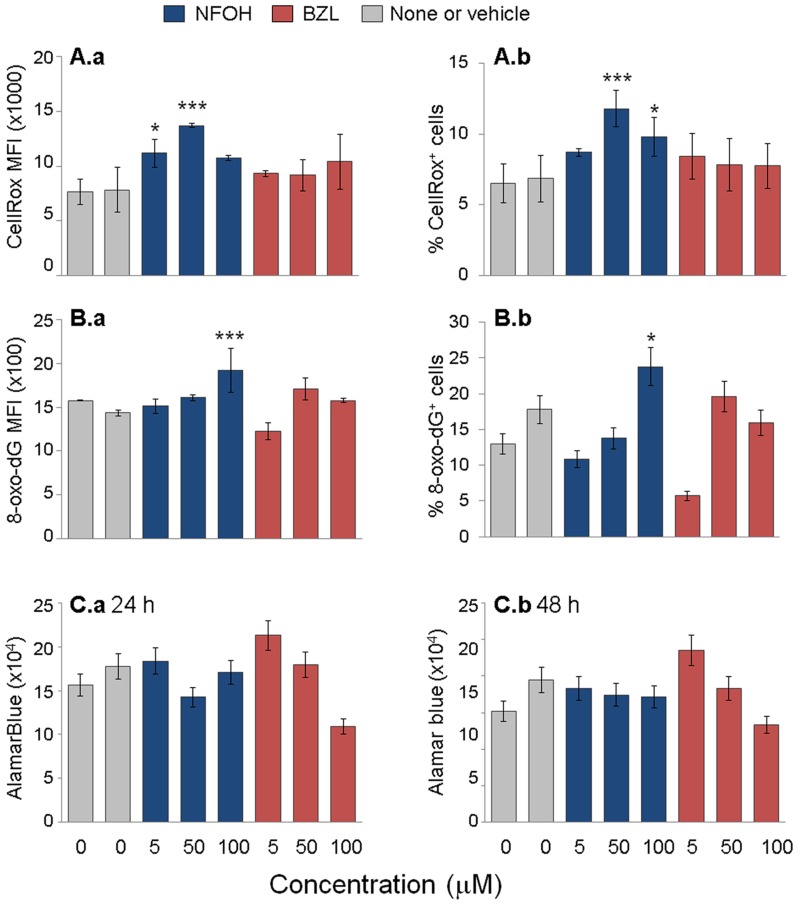
Hepatocytes dose response to NFOH and BZL. HepG2 cells (7.5×10^4^/well) were incubated in presence of 5–100 µM NFOH or BZL for 24 h. Cells incubated with vehicle solution (10% DMSO) were used as controls. Shown are flow cytometry evaluations of (**A**) CellROX Green oxidation by intracellular ROS and (**B**) 8-oxo-dG levels as an indicator of DNA damage. The mean fluorescence intensity (MFI) of CellROX (**A.a**) and 8-oxo-dG (**B.a**) as an indicator of the extent of ROS production and DNA damage, respectively; and the mean percentage of CellROX^+^ (**A.b**) and 8-oxo-dG^+^ (**B.b**) cells was determined. (**C**) HepG2 cells were incubated with 5–100 µM NFOH or BZL as above. Cell survival was determined at 24 h (**C.a**) and 48 h (**C.b**) post-treatment by an AlamarBlue assay. Data are presented as mean ± S.D. and derived from two independent experiments (triplicate observations per treatment per experiment). Significance is shown as *p<0.05, **p<0.01, ***p<0.001 (vehicle control-versus-treated).

We first evaluated the dose response of the HepG2 cell line to NFOH and BZL. Hepatocytes express cytochrome P450 isoforms, including Cyp2E1 that elicit ROS generation under stress conditions. Additionally, impairment of mitochondrial permeability transition, fatty acid β-oxidation, and inhibition of mitochondrial respiration are all potential mechanisms of ROS generation under stress conditions. We noted a 40–75% increase in CellRox fluorescence (detects intracellular ROS, (Fig. 1A.a) in 27–72% (Fig. 1A.b) of the HepG2 cells treated with 5–100 µM NFOH. The maximal increase in ROS generation was observed when HepG2 cells were treated with 50 µM NFOH treatment. The 8-oxo-2′-deoxyguanosine(8-oxo-dG) is the major product of DNA damage and concentrations of 8-oxo-dG within a cell are used as a measurement of oxidative stress. We noted an up to 33% increase in 8-oxo-dG levels (Fig. 1B.a) in 23% (Fig. 1B.b) of the HepG2 cells treated with increasing concentrations of NFOH. Despite the increase in ROS and DNA damage biomarkers, cell viability, measured by AlamarBlue assay, was not significantly altered by NFOH treatment for 24 h (Fig. 1C.a) or 48 h (Fig. 1C.b). In comparison, HepG2 cells treated with increasing concentrations of BZL showed no statistically significant increase in ROS generation and DNA damage (Fig. 1A&B); however, a cytotoxic response to increasing concentrations of BZL was noted (Fig. 1C). The maximal cytotoxicity (33% cell death) was observed when cells were treated with 100 µM BZL for 24 h (Fig. 1C.a) or 48 h (Fig. 1C.b). Together, these data suggest that NFOH (5–100 µM) induces a mild stress response in hepatopcytes, while BZL at 100 µM concentrations is cytotoxic and causes cell death.

### NFOH does not cause short- or long-term liver toxicity in mice

We used a well-established experimental model of Swiss mice for assessing the *in vivo* cytotoxicity properties of NFOH. We have previously demonstrated NFOH activity against *T. cruzi* in Swiss mice [11]. These outbred mice display a broader response to drugs than is observed in in-bred C3H/HeN, C57BL/6 and Balb/c mice [19].

**Figure 2:**
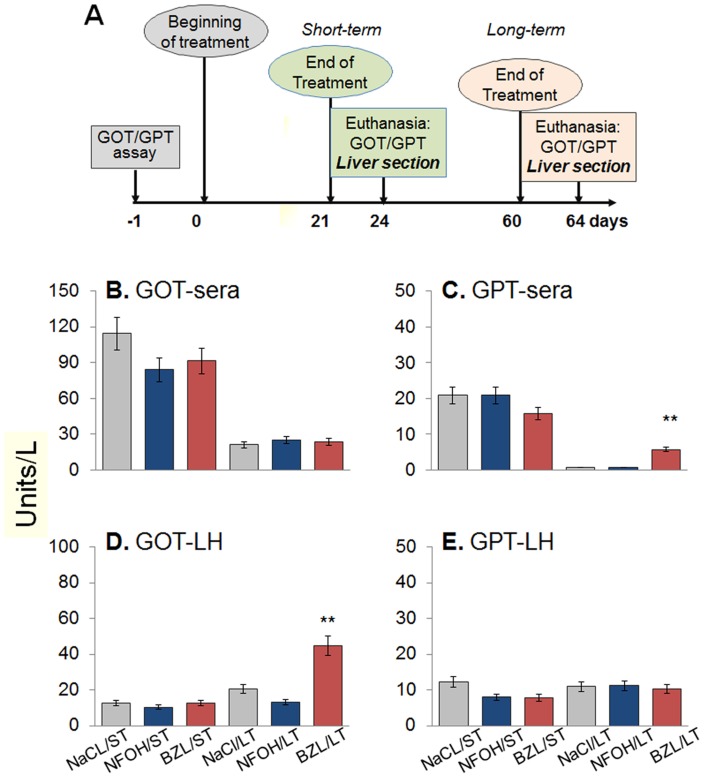
(A) Experimental design for the toxicity studies in mice. Mice received an oral dose of 150 mg/kg/day of NFOH, BZL or the vehicle (9% NaCl - 5%Tween-80) once a day for 6 days per week. Mice were treated for 21 or 60 days to determine the toxicity of short-term (ST) and long-term (LT) treatment, respectively. (**B–D**) **Liver toxicity of anti-parasite drugs.** Glutamate oxaloacetate transaminase (B&D) and glutamate pyruvate transaminase (C&E) activities in mice sera (B&C) and liver homogenates (D&E) after ST and LT treatment with NFOH and BZL were determined as an indicator of liver injury. Mice treated with vehicle only were used as controls. Data in figures 2–7 are presented as mean ± S.D. (n≥9 mice per group, triplicate observations per mouse). Significance is shown as *p<0.05, **p<0.01, ***p<0.001 (vehicle control-versus-treated).

We measured elevation in GOT and GPT activities as a general biochemical marker of liver injury after 21 d (ST) and 60 d (LT) treatment with NFOH (controls: vehicle solution). We observed no significant difference in GOT and GPT activities in the sera of mice treated with NFOH or vehicle for the ST and LT period (Fig. 2B&C). Likewise, liver levels of GOT and GPT activities in mice treated with NFOH or vehicle for ST and LT were not statistically different, and comparable to those noted in normal controls (Fig. 2D&E). In comparison, mice treated with BZL for LT exhibited a >5-fold (p<0.05) increase in the sera levels of GPT activity and liver levels of GOT activity (Fig. 2C&D). These data suggest that NFOH is not hepatotoxic, and its treatment for ST or LT is safe. In comparison, LT treatment with BZL was hepatotoxic and caused chronic cellular injury.

### Phagocyte activation and oxidative damage in response to NFOH

The host defense response to NFOH or BZL can result in activation of macrophages and neutrophils that produce oxidative burst, nitric oxide (^•^NO), and HOCl supported by induction of NADPH oxidase, inducible nitric oxide synthase (iNOS) [23]–[25], and MPO [26], respectively. The cytotoxicity of reactive oxygen species (ROS) results in oxidation of cell constituents, including proteins, lipids, and DNA, which lead to deterioration of cellular structure and function. Additionally, ^•^NO reacts with O_2_^•−^ and forms peroxynitrite (ONOO^−^) and peroxynitrous acid (ONOOH) that cause increased protein 3-nirotyrosine (3NT) formation [20], [23], [27].

**Figure 3:**
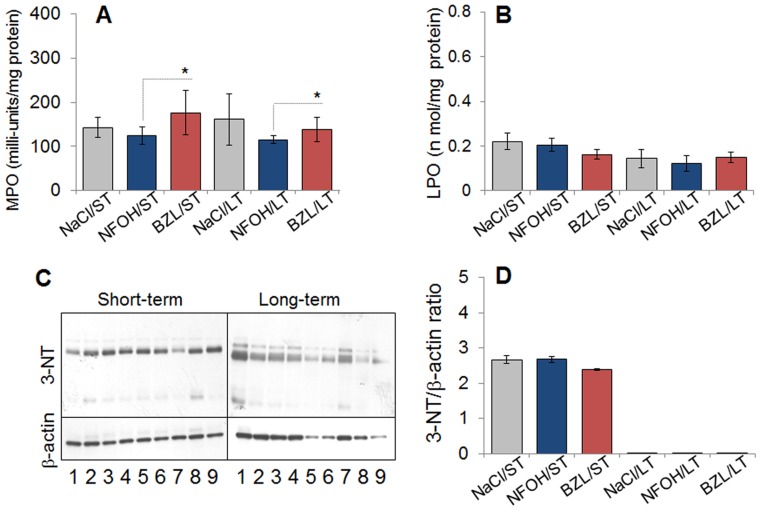
NFOH causes short-term phagocytic activation and oxidative damage. Mice were treated with NFOH and BZL as in Fig. 1, and liver tissue homogenates were prepared. (**A**) Myeloperoxidase activity was measured as a biomarker of phagocyte activation. (**B**) Lipid hydroperoxides levels were evaluated as a marker of oxidative stress. (**C&D**) Liver homogenates were subjected to Western blotting (C) with anti-3-nitrotyrosine antibody (Control: anti-β-actin antibody). Densitometry quantitation of 3-NT band intensity, normalized to β-actin, is presented in panel D.

We investigated host defense responses to NFOH and BZL by measurement of MPO activity and oxidative/nitrosative stress. The level of MPO activity in liver homogenates of mice treated with NFOH for ST or LT was not statistically different when compared to that noted in mice given vehicle only, and was within the basal-level range (100–150 milli-units/mg protein). The BZL-treated mice exhibited a 20–40% increase in liver level of MPO activity when compared to that noted in NFOH-treated mice (Fig. 3A). LPO refers to highly reactive hydroperoxides of saturated and unsaturated lipids, formed by oxidation [28]. NFOH and BZL treatment for ST or LT resulted in no significant increase in the liver levels of LPO formation in comparison to those in control mice treated with vehicle solution (Fig. 3B). The basal level of LPO (<0.25 n mol/mg protein) in all mice, irrespective of ST or LT treatment with NFOH, BZL or vehicle was within the lowest detection range. The polypeptide-bound 3-NT residues, formed by peroxynitrite attack, were monitored by Western blotting. These data showed that the 3-NT level in liver homogenates of mice given ST NFOH or BZL treatment was increased by ∼2.5-fold when compared to those in normal controls, and were similar to those noted in mice given vehicle only (Fig. 3C&D). The 3-NT contents normalized to β-actin were unchanged after LT exposure to NFOH, BZL, or vehicle in treated mice (Fig. 3C&D). Overall, the data presented in Fig. 3 suggested that treatment of mice with NFOH or BZL caused a short-term increase in nitrosative stress that was likely a placebo effect, and, in general, both anti-parasitic drugs did not elicit long-term liver injury by phagocyte activation and oxidative damage in mice.

### NFOH does not cause chronic liver inflammation

**Figure 4:**
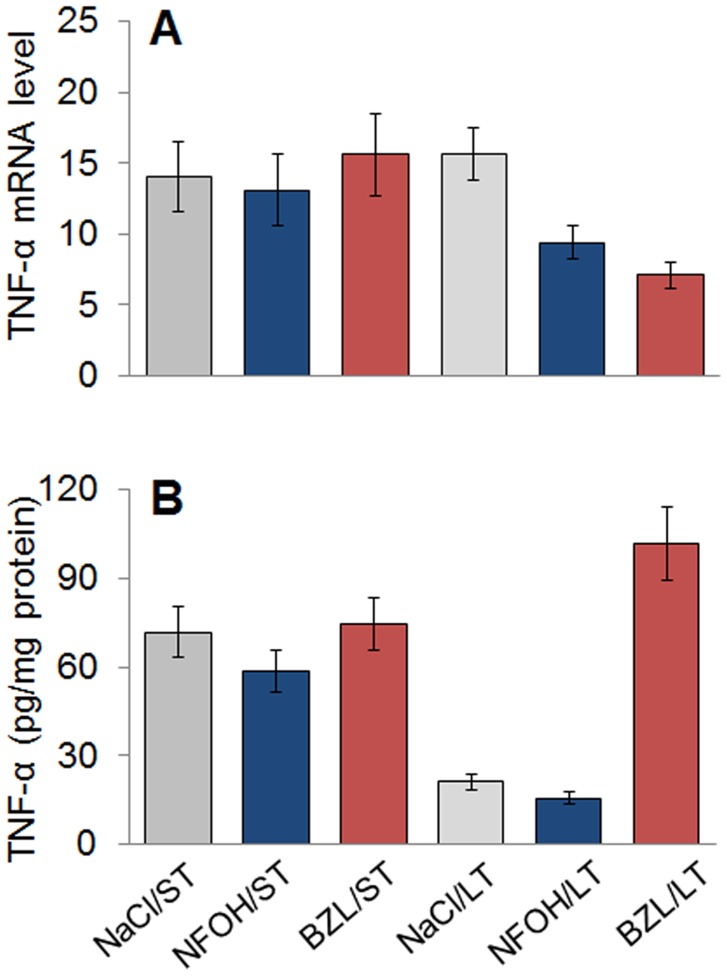
Liver inflammatory cytokine response to treatment with anti-parasite drugs. Liver tissues were harvested after ST and LT treatment with NFOH, BZL, and vehicle, as detailed in Fig. 1. (**A**) Shown are the relative changes in gene expression for TNF-α, determined by real time RT-PCR. Results were normalized to GAPDH mRNA. (**B**) TNF-α levels in tissue homogenates were determined by an ELISA.

Next, we determined the effects of NFOH and BZL treatment on liver inflammation. Mice treated with NFOH, BZL or vehicle for ST exhibited a 13–15-fold increase in TNF-α mRNA expression when compared to that noted in normal (untreated) controls (Fig. 4A). The increase in TNF-α expression was also reflected by increased levels of TNF-α protein in liver homogenates of mice treated with NFOH, BZL or vehicle only for ST (range: 58–74-pg/mg protein, Fig. 4B). When given for LT, the NFOH-induced increase in TNF-α mRNA and protein level was decreased by >4-fold, when compared to that noted after ST NFOH treatment and similar to that noted in controls (Fig. 4A&B). Mice given BZL treatment for LT exhibited a 2-fold decline in TNF-α mRNA and a 30% increase in TNF-α protein level when compared to that noted after ST BZL treatment (Fig. 4A&B).

**Figure 5:**
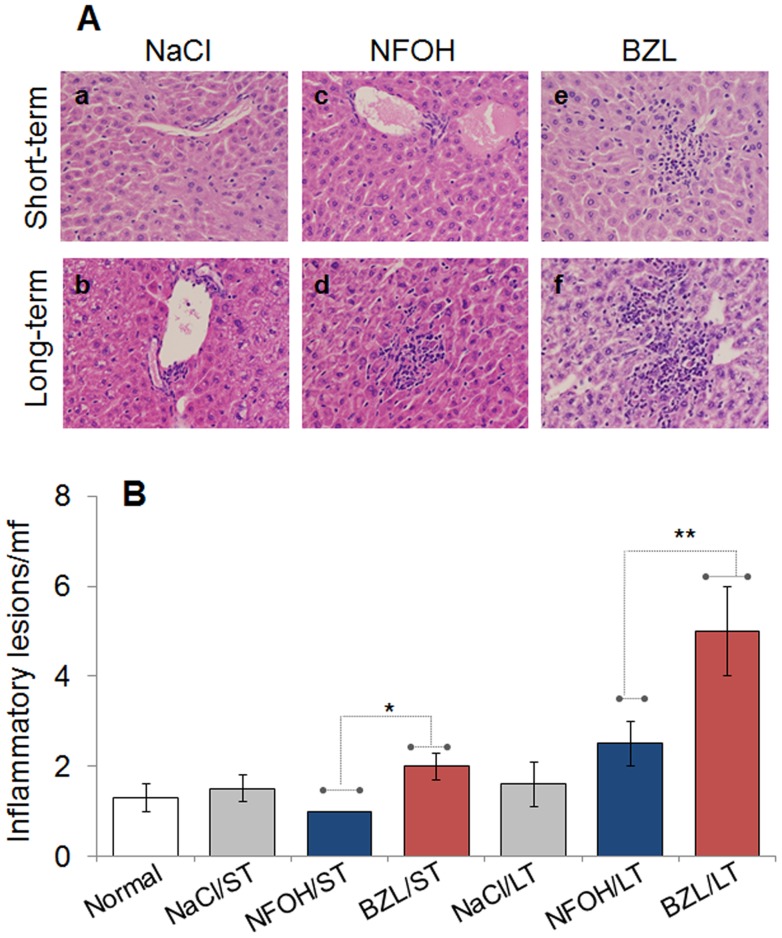
NFOH does not cause chronic liver inflammation. Paraffin-embedded liver tissue sections (5 µm) were submitted to H&E staining (blue: nuclear, pink: muscle/cytoplasm). (**A**) Shown are the representative images (magnification: 40×) of liver tissue-sections from mice treated with vehicle (a&b), NFOH (c&d), and BZL (e&f) for short-term (a,c,e) and long-term (b,d,f). (**B**) Inflammatory infiltrate in liver tissue sections (3-sections/mouse, n>3/group) was scored as described in Materials and Methods.

Histological studies showed that ST treatment with NFOH and BZL resulted in a mild increase in liver inflammatory infiltrate, extensive inflammatory lesions (histological score: II–III) being detected in BZL-treated mice followed by vehicle- and NFOH-treated mice (Fig. 5A.a,c,e & 4B). Upon LT treatment, liver inflammatory lesions were noted to be in the order of BZL>NFOH ≥ vehicle. Focal lesions with 0–2 inflammatory foci per microscopic field (histological score: II–IV) were primarily noted in the livers of mice after LT treatment with NFOH or vehicle solution (Fig. 5A.b,d & 5B). The LT treatment with BZL resulted in widespread inflammation in liver, evidenced by finding of >4-inflammatory foci/mf, extensive and diffused inflammation associated with severe tissue necrosis, interstitial edema, and loss of integrity (histological score: III–V, Fig. 5A.f & 4B). Overall, the data presented in Figs. 4&5 suggested to us that NFOH, BZL and vehicle caused a short-term increase in liver levels of TNF-α and inflammatory infiltrate that was likely a placebo effect. When used for LT, NFOH was well-tolerated, while LT treatment with BZL resulted in extensive tissue inflammation in mice.

### Liver remodeling and lipid deposition in response to NFOH

**Figure 6:**
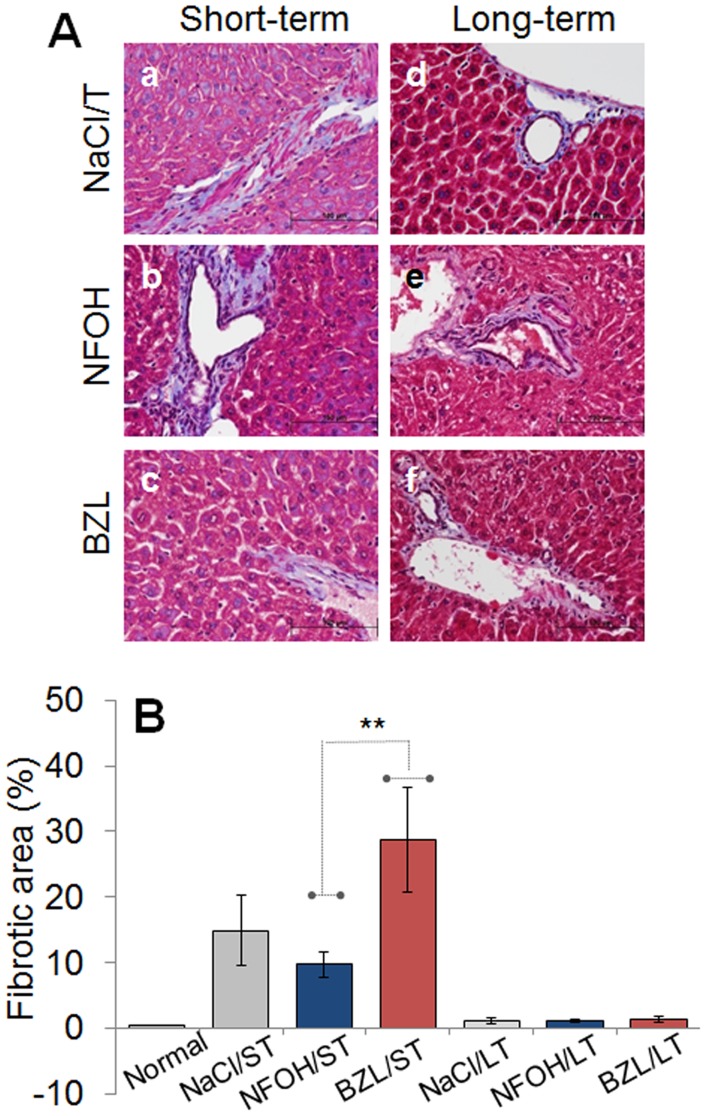
Liver remodeling in response to NFOH treatment. Mice were treated with vehicle solution (a&d), NFOH (b&e) or BZL (c&f) for a short-term (a,b,c) and long-term (d,e,f). (**A**) Representative micrographs of liver tissue-sections stained with Masson's Trichome (magnification: 400×, scale bar: 100 µm) are shown. (**B**) Tissue sections (3-sections/mouse, n>3/group) were scored for fibrosis as described in Materials and Methods.

**Figure 7:**
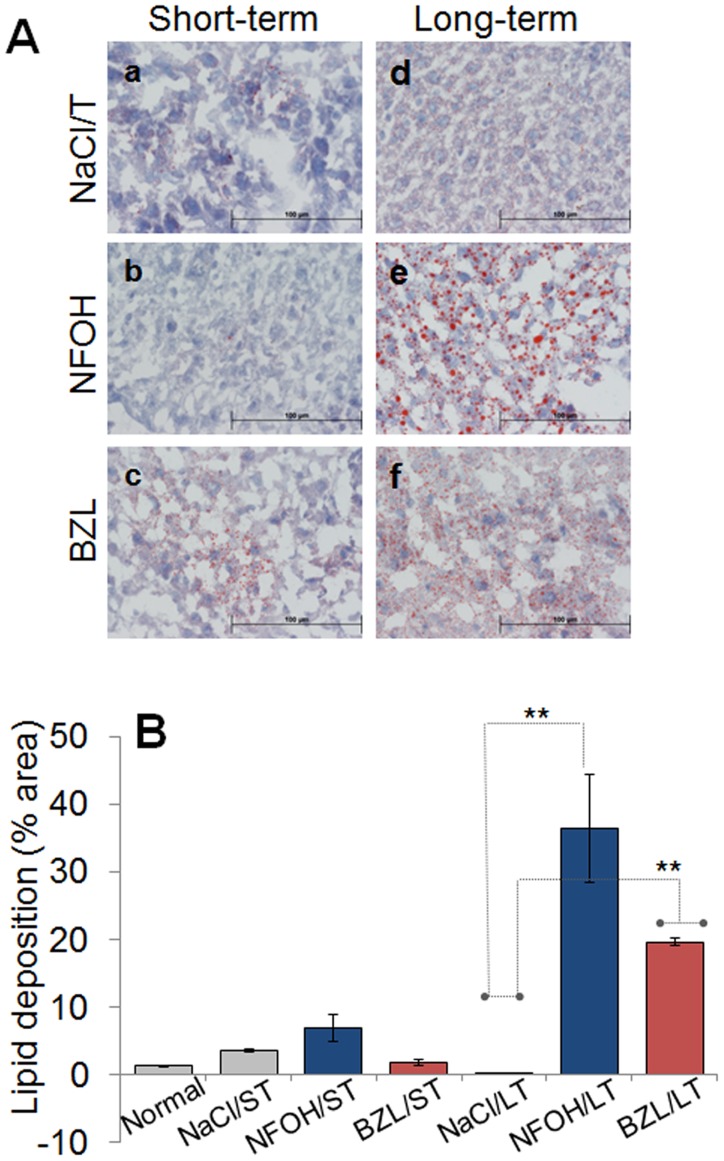
NFOH does not cause metabolic stress. Mice were treated for ST (a,b,c) and LT (d,e,f) with vehicle solution (a&d), NFOH (b&e) or BZL (c&f). (**A**) Liver deposition of lipids, an indicator of metabolic dysfunction, was determined by Oil Red O staining. Shown are the representative micrographs (magnification: 600×). (**B**) Tissue sections (3-sections/mouse, n>3/group) were scored for lipid deposition, as described in Materials and Methods.

ROS and inflammatory mediators have been suggested to promote tissue remodeling and dysfunction through diverse mechanisms [23], [29]. We performed histological staining of the liver tissue sections with Masson's Trichrome and oil red O, respectively, for the detection of collagen (Fig. 6) and lipid droplets (Fig. 7). Our data showed the ST with NFOH resulted in a mild degree of collagen deposition in <10% of the tissue area (histological score: II, Fig. 6A.b & 6B, p<0.01) that was significantly lower than that noted in mice given vehicle or BZL treatment. The BZL-treated mice exhibited an up to 30% fibrotic area (histological score: II–III) in liver tissue (Fig. 6A.c & 6B). Very few collagen lesions (<10% fibrosis, histological score: 0–I) indicative of liver remodeling were noted after LT treatment with NFOH, BZL or vehicle solution (Fig. 6A.d–f & 6B).

The extent of lipid deposition, an indicator of mitochondrial dysfunction, was not significantly different after ST treatment with NFOH, BZL or vehicle (Fig. 7A.a–c & 7B). LT treatment with NFOH and BZL resulted in extensive and uniformly scattered lipid droplets of variable size in the liver tissue of mice (20–35% of total area; histological score III–IV) than was noted in vehicle-treated mice (Fig. 7A.d–f & 7B, p<0.01). The extent of lipid deposition appeared to be high in NFOH-treated mice. Together, the results presented in Figs. 6&7 suggest that BZL resulted in mild-moderate acute remodeling of the liver that was replaced by extensive lipid deposition after LT treatment. In comparison, ST treatment with NFOH was not pro-fibrotic, and long-term treatment with NFOH resulted in minimal remodeling and a degree of metabolic dysfunction in the liver of treated mice.

## Discussion

BZL and NFX, drugs that provide the only line of therapy against acute *T. cruzi* infection, were released to the market without extensive testing for possible adverse effects, which have been reported over the four decades that these drugs have been in use. It was confirmed that NFX has more severe secondary effects than does BZL; these range from alterations of the cellular immune responses [30], peripheral nervous system toxicity, and testicular and ovarian damage to mutagenic effects [17], [31]–[34]. In clinical practice, it is recommended to interrupt anti-*T. cruzi* treatment when the adverse effects of BZL are detected in adult patients [35]. In contrast, children and newborns show a better tolerance to BZL [36], [37].

Different strategies with the overall aim of finding a cure for Chagas disease are currently under investigation. The major challenges to testing and implementation of therapeutic use of the currently available drugs (BZL and NFX) include the resistance of many of the naturally occurring parasite isolates (e.g. Colombiana) [9] and low efficacy of treatment during indeterminate and chronic phase of disease [6]–[9]. Moreover, additional challenges are faced in immuno-suppressed chagasic patients that become recipients of transplanted organs or are HIV co-infected. The immuno-suppressed patients present a short window of time when they should be treated with anti-parasite drugs. Otherwise, parasite recurrence results in severe acute infection and organ failure. Due to high toxicity concerns, BZL and NFX are not always recommended for treatment of immuno-suppressed patients [38]. In this scenario, NFOH has emerged as a promising compound for its anti-*T. cruzi* activity, both *in vitro* and *in vivo*, and its favorable pharmacological properties [39], [40]. NFOH, derived from hydroxymethyl substitution at the primary amide of nitrofurazone [15], also has a higher solubility in water than does NF and BZL, which likely would facilitate its oral administration. In a murine model of acute *T. cruzi* infection, NFOH was highly effective in controlling parasitemia evidenced by the observation that infected mice, after NFOH treatment, exhibited no signal for parasite DNA by a highly sensitive PCR approach, and sero-converted with depletion of anti-parasite antibodies [11].

NFOH is a derivative of nitrofurazone (NF). NF is highly toxic and shown to result in single strand DNA breaks [41] and oxidative DNA damage [42], and is considered to be potentially carcinogenic [13]. Considering the high toxicity of NF, it is important to evaluate the toxicity of NFOH *in vitro* and *in vivo* before it is recommended for treatment of *T. cruzi* infection in humans. Accordingly, the present study was designed to examine the adverse effects of NFOH treatment in HepG2 cells and ST and LT treatment of NFOH in mice. We focused on examining the effects of NFOH on hepatocytes and liver because the liver is the main detoxifying organ in mammals. Metabolism of xenobiotics in the liver involves phase I and phase II reactions that add hydroxyl and methyl groups, respectively, to a given compound. NFOH is a nitrofurazone with an *N*-hydroxymethylation at the primary amide and was anticipated to cause significantly reduced toxicity [11].

Our *in vitro* studies evaluating the dose response of HepG2 cells clearly demonstrated that NFOH at higher concentrations (50–100 µM) induced mild stress as was evidenced by the observation of ROS production and DNA damage (Fig. 1A&B). However, the NFOH-induced stress was controlled as we observed no cell death in NFOH-treated HepG2 cells. Under similar conditions, cytotoxicity of BZL was evidenced by induction of cell death in 33% of the cells (Fig. 1C). Others have shown the nifurtimox and BZL inhibited DNA and protein synthesis in hepatocytes [43]. In murine studies, the selected doses for toxicity evaluation were the same as those that we have previously tested in mouse models of acute and chronic *T. cruzi* infection. We included mice treated with BZL and vehicle (NaCl/Tween-80) as controls. Our data showed a moderate increase in sera levels of GOT and GPT (Fig. 1) after ST and LT treatment with NFOH that was similar to that noted in mice treated with vehicle only. Further, MPO activity and LPO production, measured as markers of neutrophil activation and macrophage oxidative burst, were present at the lowest limits of detection in all mice given NFOH or vehicle throughout the treatment schedule (Fig. 3A&B). These data, along with the observation of no significant change in TNFα mRNA and protein level (Fig. 4) suggested to us that NFOH did not induce hepatic stress associated with cellular injury, oxidative stress, and innate immune cell activation *in vitro* or *in vivo* after ST or LT treatment. Our observation of a >5-fold increase in hepatic levels of MPO activity and TNF-α expression in the livers of mice within 3 d after a single dose treatment with 2,3,7,8-tetrachlorodibenzo-p-dioxin (TCDD, 20-µg/Kg/100 µl peanut oil) in other studies suggest that when compared with TCDD effects, NFOH has no acute liver toxicity. The notion of NFOH being liver-safe is also supported by the observation of a higher degree of GOT release (5-fold), MPO activity (20–40%), and TNF-α protein levels (up to 20%) in BZL-treated mice in this study (Figs. 2–4). Others have also shown the BZL toxicity by alterations in mitochondrial function in liver of treated rats [44].

We corroborated the biochemical findings (Figs. 1–4) of NFOH safety by histological observations of tissue inflammatory infiltrate, fibrosis, and lipid deposition in the livers of mice treated for ST or LT with NFOH, and compared the findings with BZL-treated mice (Figs. 5–7). NFOH-treated mice consistently exhibited none-to-low levels of inflammatory infiltrate and fibrotic lesions in liver tissue sections that were similar to or lower than was noted in vehicle-treated mice (Figs. 5–7). The extent of liver inflammatory infiltrate was significantly higher in BZL-treated mice, especially after LT treatment (Figs. 5). Likewise, BZL-treated mice exhibited an acute liver fibrosis (Fig. 6) similar to what we have noted in TCDD-treated mice (unpublished data). The extent of lipid deposition in liver tissue after LT treatment with BZL and NFOH was comparable to lipid deposits provoked within three days after treatment with a single dose of TCDD. Though not withdrawn from market, BZL has been demonstrated to be more toxic than NFOH with regard to elicitation of liver inflammatory responses, fibrosis, and a cellular dysfunction at mitochondrial level, both in our data presented in this study and other published reports [17], [33]. We surmise that NFOH only causes a mild transient hepatic injury similar to that caused by vehicle treatment only in mice. Our results encourage further research on carcinogenicity, and mechanism of action of NFOH to address its potential safety for human use as an anti-parasite therapy.

In conclusion, our results showed that ST and LT treatment with NFOH elicited similar or lower levels of liver oxidative stress, inflammation and tissue remodeling responses, when compared to that noted by a similar regimen of treatment with vehicle only. In comparison, BZL that has been used for the treatment of human chagasic patients was more toxic and induced chronic inflammation and liver injury. Our data provide the impetus for future studies focusing on further characterization of anti-parasite efficacy, toxicity, and carcinogenicity of NFOH, aiming to determine its potential safety to be considered as a drug candidate for the treatment of Chagas disease.
